# Recent Advances in Cancer Vaccines: Challenges, Achievements, and Futuristic Prospects

**DOI:** 10.3390/vaccines10122011

**Published:** 2022-11-25

**Authors:** Madhu Gupta, Abhishek Wahi, Priyanka Sharma, Riya Nagpal, Neha Raina, Monika Kaurav, Jaydeep Bhattacharya, Sonia M. Rodrigues Oliveira, Karma G. Dolma, Alok K. Paul, Maria de Lourdes Pereira, Polrat Wilairatana, Mohammed Rahmatullah, Veeranoot Nissapatorn

**Affiliations:** 1 Department of Pharmaceutics, School of Pharmaceutical Sciences, Delhi Pharmaceutical Sciences and Research University, New Delhi 110017, India; 2 Department of Pharmaceutics, KIET Group of Institutions Delhi-NCR, Meerut Road (NH-58), Ghaziabad 201206, India; 3 School of Biotechnology, Jawaharlal Nehru University, New Delhi 110067, India; 4 CICECO-Aveiro Institute of Materials, University of Aveiro, 3810-193 Aveiro, Portugal; 5 Hunter Medical Research Institute, New Lambton Heights, NSW 2305, Australia; 6 Department of Microbiology, Sikkim Manipal Institute of Medical Sciences, Sikkim Manipal University, Gangtok 737102, India; 7 School of Pharmacy and Pharmacology, University of Tasmania, Hobart, TAS 7001, Australia; 8 Department of Medical Sciences, University of Aveiro, 3810-193 Aveiro, Portugal; 9 Department of Clinical Tropical Medicine, Faculty of Tropical Medicine, Mahidol University, Bangkok 10400, Thailand; 10 Department of Biotechnology & Genetic Engineering, University of Development Alternative, Lalmatia, Dhaka 1207, Bangladesh; 11 School of Allied Health Sciences and World Union for Herbal Drug Discovery (WUHeDD), Walailak University, Nakhon Si Thammarat 80160, Thailand

**Keywords:** cancer, immunotherapy, vaccine, nucleic acid, peptides, antigen

## Abstract

Cancer is a chronic disease, and it can be lethal due to limited therapeutic options. The conventional treatment options for cancer have numerous challenges, such as a low blood circulation time as well as poor solubility of anticancer drugs. Therapeutic cancer vaccines emerged to try to improve anticancer drugs’ efficiency and to deliver them to the target site. Cancer vaccines are considered a viable therapeutic technique for most solid tumors. Vaccines boost antitumor immunity by delivering tumor antigens, nucleic acids, entire cells, and peptides. Cancer vaccines are designed to induce long-term antitumor memory, causing tumor regression, eradicate minimal residual illness, and prevent non-specific or unpleasant effects. These vaccines can assist in the elimination of cancer cells from various organs or organ systems in the body, with minimal risk of tumor recurrence or metastasis. Vaccines and antigens for anticancer therapy are discussed in this review, including current vaccine adjuvants and mechanisms of action for various types of vaccines, such as DNA- or mRNA-based cancer vaccines. Potential applications of these vaccines focusing on their clinical use for better therapeutic efficacy are also discussed along with the latest research available in this field.

## 1. Introduction

Cancer is associated with high mortality and prevalence rates despite extensive research on its etiology, diagnostics, novel therapeutic biomarkers and targets, as well as technological breakthroughs [1]. In the United States, cancer is the second leading cause of death, and in the years to come, it is expected to surpass deaths from heart disease. According to a figure reported by the WHO in 2015, 7.6 million people worldwide died due to cancer alone [2]. Thus, solid investment must be made into finding new therapeutic options that can prevent and/or treat this global health problem. Cancer vaccines offer an option of immunotherapy that can “educate” the immune system to recognize and eliminate cancer cells. The first vaccine was developed in 1796 by Dr. Edward Jenner against smallpox (Variola). Since then, multiple vaccines have contributed to control or even eliminate epidemic- and pandemic-prone infections, in both humans and animals. Numerous cancer studies have been conducted in both animal models and xenografts from human volunteers with the objective of developing vaccines to treat cancer by stimulating the immune system to identify and eradicate tumors [3]. Finding and targeting suitable epitopes or antigens expressed only by cancer cells is a crucial stage for the development of a cancer vaccine [4]. However, the development of cancer vaccines proved even more challenging as promising pre-clinical findings have not translated into the clinic. Numerous factors may contribute to these failures, including a poor understanding of the tumor biology and its immune-suppressive tumor microenvironments (TME), the weak response of T-cells, inadequate vaccine formulations, adjuvants, and choosing the right patients to target [5]. Sipuleucel-T (Provenge^®^) is a vaccine composed of patients’ own stimulated dendritic cells used to treat metastatic castration-resistant prostate cancer in a small number of largely asymptomatic patients, and is the first of only three vaccines for cancer treatment that the FDA has approved so far [6]. The other two FDA-approved cancer vaccines are live BCG (Bacillus Calmette–Guérin) against early-stage bladder cancer and Talimogene laherparepvec^®^ against melanoma. Four other formulations were approved as preventive cancer vaccines, namely against HPV (Human Papillomavirus) and HBV (Hepatitis B virus), known to cause cervical cancer and head and neck cancer, and liver cancer, respectively. However, recent advances in immunotherapy show the potential to create more successful cancer vaccines. Notwithstanding, we need to extend our understanding of the biology of tumors, particularly of their unique targets or epitopes, and biomarkers that can help in early diagnosis, prognosis, or response to cancer therapy. Tumor antigens have limited immunogenicity due to their endogenous nature, in contrast to antigens from foreign pathogens that are targeted by conventional vaccines [7]. Several tumor neoantigens (unique antigens that result from certain mutations in tumor DNA) have been discovered as a result of the accessibility and affordability of high-throughput sequencing methods. In fact, advanced medical technologies such as genomics or proteomics have been key to the recent breakthroughs and developed of novel diagnostics and treatment options. It is not, therefore, surprising that vaccines and cancer therapies are rapidly evolving and becoming extraordinarily complex, multifaceted, and subjected to rapid changes as the technology evolves. The development of effective cancer vaccines remains elusive and extraordinarily challenging, as cancer cells more closely resemble normal, healthy cells. Moreover, it is now clear that as each individual tumor can be to some degree unique, with its own neoantigens and antigens, we need to invest into more sophisticated, personalized, and targeted approaches.

Cancer vaccines can be classified into four groups according to preparation techniques: vaccines made from nucleic acids, cells, viruses, and peptide-based vaccines (Table 1) [8]. RNA- and DNA-based vaccines, which contain the antigen-carrying group of pathogens and the encoding gene, are examples of nucleic acid vaccines. Cell-based cancer vaccines are vaccines that employ entire cells as antigen carriers. For the treatment and prevention of some tumors, virus-based cancer vaccines primarily use viruses as vectors, such as the vaccines against HPV (Cervarix^®^, Gardasil^®^, Gardasil-9^®^) [9,10]. Adjuvants must be included in peptide-based vaccines since they are frequently less immunogenic. Cancer DNA vaccines are closed-circular DNA plasmids that encode tumor-associated antigens (TAAs) or immunomodulatory compounds to elicit tumor-specific responses. Similarly, in vitro-produced mRNA vaccines may encode antigens and, after internalization, express proteins to elicit an immune response [11,12]. Therapeutic DNA vaccines are considered the most promising strategy to activate the immune system against cancer [13]. However, in a step further, personalized neoantigen (or DNA) vaccines can be more successful against cancer. In contrast to normal-yet-overexpressed proteins, tumors also express unique targets or epitopes resulting from mutations that are often designated as neoantigens (“new antigens”) and are expressed only by tumor cells and may even be as specific as they are expressed only by a very small cohort of patients with this type of tumor. With personalized neoantigen vaccines, it is possible to direct immune responses precisely against patients’ tumor cells without affecting healthy cells, hence preventing deleterious side effects. Several types of neoantigen vaccines are being investigated, both alone and in combination with other treatments, in several cancer types. Combining cancer vaccines with different immunotherapies or regular treatments has been proven to be a successful tactic for combating tumor resistance and enhancing clinical outcomes [14]. Research on cancer vaccines has been greatly aided by the continuous investigation of tumor biology, immunological mechanisms, and numerous innovative medical and nanotechnologies. A vaccination platform should be chosen after considering a number of considerations, such as, for example, the selection of the right tumor antigen for stimulation of effective T-cells, maximizing the antigen concentration on dendritic cells (DCs), the morphology of the cells, and the tumor size, as well as the amount of time and resources needed to prepare personalized vaccines. Nucleic acid vaccines would be the most time-efficient option for various metastatic diseases. Meanwhile, combination therapy may also be employed in the vaccines’ development process to prevent further illness exacerbations and to create an environment that helps the immune system respond more effectively [15]. This review systematically describes the latest developments in various types of vaccines and antigens for anticancer therapy, mechanisms of cancer vaccines, newly emerging cancer vaccine adjuvants, and nanocarrier systems as cancer adjuvants.

## 2. Current Vaccines and Antigens for Cancer Treatment 

A vaccine is created to develop specific immunity against a particular disease or infection. The purpose of cancer immunotherapy is to activate the immune system so that it can identify and eliminate cancer cells. Anticancer immunotherapies are classified as either “passive” or “active” based on their ability to (re-)activate the host immune system against malignant cells. Tumor-targeting monoclonal antibodies (mAbs) and adoptively transferred T-cells (among other approaches) are considered passive forms of immunotherapy because they have intrinsic anticancer activity [21,22]. Antigen-specificity is an alternative classification of immunotherapeutic anticancer regimens. While tumor-targeting mAbs are widely regarded as antigen-specific interventions, immunostimulatory cytokines or checkpoint blockers activate anticancer immune responses with unknown (and generally broad) specificity [23,24]. Commonly used vaccines and antigens are listed in Table 2.

### 2.1. Antitumor Response of Therapeutic Vaccine

**Figure 1:**
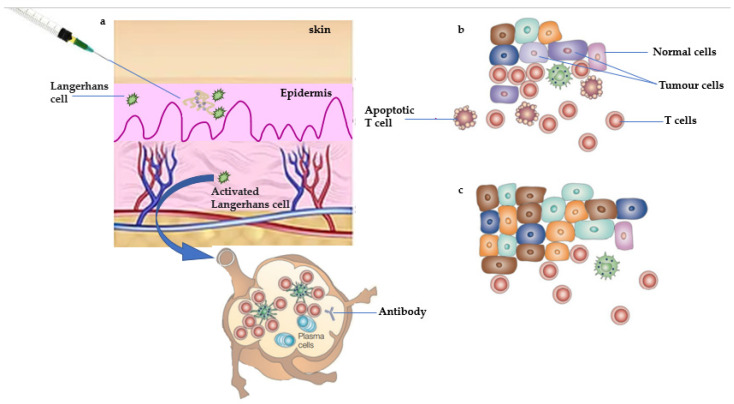
Manipulation of antitumor immune responses by therapeutic vaccination. (**a**) Therapeutic vaccines are administered after the tumor is diagnosed, at the time of interactions between the tumor and the immune system that correspond to part (**c**). Therapeutic vaccines boost immunity against minimal residual disease and prevent the outgrowth of metastases shown in parts (**b**,**c**). A vaccine based on an autologous tumor was administered in an immunostimulatory preparation (with adjuvant) that can activate Langerhans cells—dendritic cells (DCs) that reside in the epidermis. Activated Langerhans cells take up the tumor antigens and traffic to the draining lymph node in which they present antigens to T-cells. B-cells are also activated, and the expected outcome is clonal expansion of tumor-specific T-cells and the production of tumor-specific antibodies. (**b**) Tumor-specific T-cells migrate to the sites of tumor metastases where they attempt to kill tumor cells that express antigens contained in the vaccine. (**c**) Metastases that continue to grow are composed of tumor cells that lack antigens recognized by T-cells and antibodies or are otherwise resistant to immune destruction.

Although many primary tumors can be surgically removed and there is often a long period of time before the tumor recurs at metastatic sites, cancer vaccines have been proposed as a therapy to elicit and/or boost antitumor immunity in patients with minimal residual disease, thereby preventing or delaying recurrence. Few vaccines have been evaluated in this ideal clinical setting [25]. So far, the majority of phase I and II studies have been conducted in late-stage disease with a relatively large tumor burden following the failure of standard therapies. Even in the best of circumstances, the ability of the immune system to overcome tumor-induced, therapy-induced, or age-induced immunosuppression will be critical to the success of therapeutic vaccines. Another factor that influences the effectiveness of therapeutic vaccines is the growth of tumor cells, which can evade the immune response for various reasons (Figure 1). The development of vaccines for melanoma patients has yielded the most clinical results of any therapeutic vaccine effort. It began with the use of cell lysates from allogeneic tumor cell lines in combination with adjuvants [26,27] or protein products shed into such cell lines [28,29]. These studies included hundreds of patients with advanced stage III or IV melanoma, many of whom had metastatic disease and had failed chemotherapy. In the case of one of these vaccines, Melacine (Corixa Corporation, Seattle, WA, USA), phase I and II trials in stage IV patients revealed a 1020% response rate (clearing of some metastatic sites) and disease stabilization in another 1020% of patients (no progression of tumors that were growing for various periods of time). Melacine was compared to a four-drug chemotherapy regimen in a multi-center phase III study, and the response rates and survival rates were the same [30]. Melacine had an advantage over chemotherapy because it is non-toxic, allowing a higher quality of life when compared to chemotherapy. As a result, Melacine is now available on prescription in Canada and is awaiting approval in the United States. Canvaxin, a similar vaccine preparation, was tested in 1000 stage IV melanoma patients and compared to an equal number of patients who received surgery and chemotherapy but did not receive the vaccine during the same time period. In this single-institution study, the vaccinated group had a small but statistically significant increase in overall survival. A multi-center phase III randomized trial is now underway to evaluate the vaccine.

DC-based vaccines [31] are the most recent advancement in cancer vaccine development. Autologous or allogeneic tumors [32], apoptotic bodies [33], tumor lysates [34], tumor RNA [35,36], and tumor DNA [37,38] can be loaded into DCs. Most of these preparations have been shown in animal models to be immunogenic and to have the potential for tumor rejection and are currently being evaluated in the clinic. Recently, the results of a phase I study of a vaccine composed of DCs loaded with messenger RNA encoding prostate-specific antigen (PSA) were published. Vaccination of prostate cancer patients with elevated levels of PSA expression induced T-cell responses against PSA in the majority of patients, and the log slope of PSA was temporarily decreased [39], possibly indicating that tumor growth was slowing.

### 2.2. Antitumor Response of Prophylactic Vaccine 

**Figure 2:**
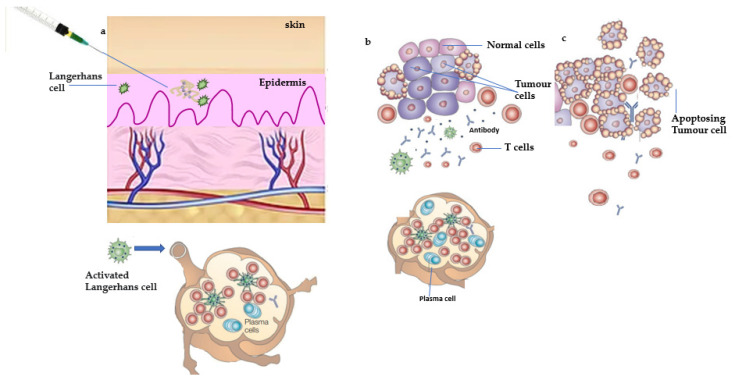
Proposed mechanism of action of prophylactic vaccine. (**a**) Prophylactic vaccination is used to manipulate antitumor immune responses. Individuals who are at high risk of developing tumors or have been diagnosed with premalignant changes in target tissues would receive prophylactic vaccines prior to the occurrence of tumors. A vaccine based on antigens expected to be expressed by the anticipated tumor is administered in an immunostimulatory preparation (with adjuvant) that can activate Langerhans cells—epidermal dendritic cells (DCs). Activated Langerhans cells transport tumor antigens to the draining lymph node, where they present antigens to T-cells. B-cells are also activated, with the expected result of clonal expansion of tumor-specific T-cells and antibody production. This clonal expansion of effector cells is followed by the generation of a pool of memory cells specific for the tumor antigen/s over time. (**b**) If a tumor grows in the future, tumor antigens that reach the draining lymph node will reactivate tumor-specific memory cells and trigger a rapid secondary immune response. This response will be distinguished by a large number of effector T-cells, a high titer of antibodies, and continuous activation of DCs at the tumor site, allowing for continuous processing and presentation of tumor antigens and further immune amplification. (**c**) Since the incipient tumor has not grown large and heterogeneous, it is easily eliminated by the prepared immune response. Furthermore, the memory compartment is expanded by this tumor-mediated boost.

Many of the potentially insurmountable issues that limit cancer vaccines’ therapeutic efficacy would not need to be addressed in the context of preventing cancer. An immune system that has been trained to recognize tumor antigens is predicted to damage the tumor before it becomes clinically visible, heterogeneous, and capable of suppressing and evading the immune response (Figure 2).

A prophylactic cancer vaccine aims to induce an adaptive primary immune response, enabling a quick and powerful secondary immune response in the event of carcinogenesis [40,41]. The first example of this approach is the Hepatitis B virus (HBV) vaccine, a virus that can lead to cirrhosis, liver cancer, and chronic hepatitis. According to early studies in Taiwanese children, the vaccination program against HBV reduced the risk of hepatocellular carcinoma by 70% [42]. The Human Papillomavirus (HPV) vaccine soon followed [42,43,44]. HPV is a family of carcinogenic, sexually transmitted viruses that can cause various neoplastic illnesses, from benign lesions to metastatic carcinomas. Furthermore, pre-approval trials have shown a very high level of vaccination efficacy (near 100%) [45]. These preventative vaccines work by eliciting an immune response to cells that have undergone malignant transformation through a process known as specific immunity to changed self-antigens [46,47]. When microbes and other foreign substances present in vaccination are administered, it alerts the host’s immune system through the display of damage-associated molecular patterns (DAMPs), which drives innate immune cells such as APCs to release cytokines required for activating T-cells [40]. A prophylactic vaccine can stimulate the development of memory T- and B-cells, which are necessary to prevent a subsequent attack or antigen exposure. When exposed again, the response is more substantial and quicker due to the proliferation of these memory cells [48]. However, many preventative vaccines require unnecessary exposure to cancer antigens, and these vaccines must be designed to ensure that antigens do not increase cancer risk [49]. Additionally, this can make it difficult for the general population to successfully accept and apply preventative cancer vaccines in medical settings. Moreover, there are safety concerns about off-target effects and toxicity of any vaccination components. Another challenge for prophylactic cancer vaccines is the immune system deterioration in aged people, as adaptive immunity is crucial to vaccine effectiveness. Hence, these issues must be addressed to develop a successful prophylactic vaccine [50]. 

**Table 2:** Current therapies as cancer vaccines and antigens [38].

Name of Vaccine/ Antigens	Type of Vaccine	Targeted Site	Combination/Route of Administration
Onyvax	Anti-idiotype vaccine	Colorectal adenocarcinoma	Either intramuscularly with the alum adjuvant or endemic with the BCG vaccine
OncoVAX	A personalized vaccination/Autologous vaccine	Stomach cancer	Not in use
Cancer VAX	Autologous vaccine	Surgery for the management of patients with stage III melanoma	Along with the BCG vaccine, another vaccine is administered
NY-ESO-1	Peptide vaccine	Stage II to IV cancer displaying the NY-ESO-1, LAGE marker LAGE, or NY-ESO-1 antigens	Endemic
11D10	Anti-idiotype vaccine	Non-small cell lung cancer in stages II or IIIA (T1-3, N1-2, M0)	Beginning 14 to 45 days following surgery
GP100 AND MART-1	GP100, MART-1, and tyrosinase peptides	Stage III or IV ocular or mucosal melanoma, or stage IIB IIC, III, or IV cutaneous melanoma	In addition to the alum adjuvant
ALVAC-CEA/B7.1	Virus antigens	Metastatic colorectal cancer	Provided together with treatment as soon as a condition is diagnosed
VG-1000 Vaccine	Autologous therapy	Carcinomas and melanomas	First-line therapy for people with newly discovered malignancies
HSPPC-96, or Oncophage	Antigens extracted from melanoma	Autologous therapy	Heat-shock protein
Sipuleucel-T	Dendritic cell vaccine	Metastases castrate-resistant cancer that is silent or barely symptomatic (hormone refractory) breast cancer	Intramuscularly
HPV Vaccine • Gardasil	Human papillomavirus (HPV)	Girl’s and women’s vulvar, vaginal, and cervical cancer	Given intramuscularly in the greater posterolateral portion of the thigh or the deltoid portion of the right forearm
Cervarix	Human papillomavirus (HPV)	Types 16 and 18 of the carcinogenic human papillomaviruses (HPV)	Three injections of 0.5 mL each into the muscle
*Other drugs*			
Thalidomide		Multiple myeloma	It is advised to take 200 mg of Thalomid once daily (in 28-day treatment cycles) orally with water, ideally just before bed and at least an hour after dinner.
Lenalidomide		Multiple myeloma	Administered orally
Bacillus Calmette–Guérin		Bladder cancer in its superficial stages, colon cancer, lung cancer, and melanoma	There are several ways to deliver Bacillus Calmette–Guerin, including intravenously, subcutaneously, directly into some tumors, intranasally, pharyngeally, or as an inhalation spray into the lungs.

Novel insights into the mechanisms and physiological processes by which the human body can identify and eliminate pre-cancerous and cancerous cells spontaneously, as well as in reaction to certain therapeutic interventions, have been reported in recent years [51,52]. Furthermore, pure, transgenic, or synthesized markers have been used in peptide-based antitumor vaccines to immunize only against a particular set of tumor-associated antigens (TAAs) and then to trigger an immune or inflammatory response against these markers. A variety of methods have been employed in recent years to discover effective antitumor immunotherapies [53,54,55,56] ranging from immunomodulatory monoclonal antibodies (mAbs), which attack CD4^+^ and CD8^+^ T-cells protein receptor cytotoxic T-lymphocyte-associated antigen (CTLA-4, also known as CD152), and the transmembrane ligand/receptor axis PD-1/PD-L1 (programmed cell death protein 1 (or CD279)/programmed cell death ligand 1), to vaccine adjuvants and precision delivery based on biomimetic formulations. Additionally, many of these immuno-technologies also activate co-stimulatory receptors found on immunological effector cell surfaces and neutralize substances generated in the TME, such as transforming growth factor β_1_ [57,58]. In a cancer treatment that includes immunotherapies such as immunostimulatory cell damage (ICD) causative agents, immunostimulatory cytokines are known to activate the immune system of cancer patients, which has been an important treatment modality. In this context, interferon alpha (IFNα) was approved for adjuvant treatment of completely resected high-risk melanoma patients and several refractory malignancies, and high-dose interleukin-2 (HDIL-2) was approved for treatment of metastatic renal cell cancer and melanoma [59]. Some of the marketed cancer immunotherapies are listed in Table 3 [17,60]. Major histocompatibility complex (MHC) components coupled to antibodies are expressed in high quantities by APCs, and have the capacity to take up and handle antigens [61]. Innate defense cells known as oligodendrocytes (DCs) were initially identified and described by Ralph Steinman in 1973 [62]. Due to their distinct characteristics and functions, DCs are by far the most significant APCs working at the juncture of innate and adaptive immunity, which causes the body to activate immunological responses. Different DC subsets have distinct differences in history and transmitter activation [63]. 

## 3. Mechanisms of Cancer Vaccines

**Figure 3:**
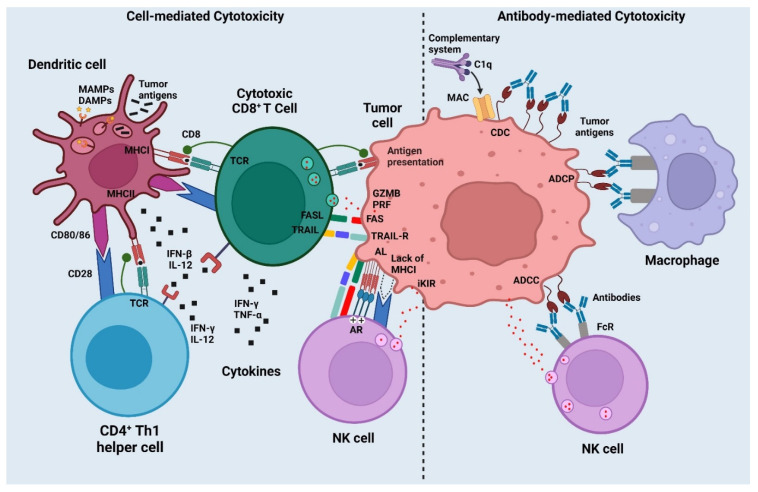
Mechanism of action of cancer vaccines (created with biorender.com).

For a therapeutic cancer vaccine, it is a prerequisite that it triggers a strong immunological reaction, precisely recognizes, and gets rid of tumor cells (primary and secondary), is antigen-specific, has minimal systemic side effects, and does not generate autoimmune responses [5]. Another consideration is that the vaccine must induce a robust immunological recall to counteract cancer cells, which is critical to attain long-term disease resolution [3]. In reality, relapses, rather than the primary tumor, have been largely blamed for the high cancer mortality rate [64]. The aim of immunotherapy-based cancer vaccines is to activate the endogenous cellular- or humoral-acquired immune system against cancer. Mostly, cancer vaccines induce the production of cancer-specific CD8^+^ T-cells that specifically recognize and kill cancer cells [65]. Tumor antigen-specific cytotoxic T lymphocytes (CTLs) recognize cancer antigen epitopes by binding to their T-cell receptor (TCR). Furthermore, CTLs via several TCR signaling pathways, such as degranulation (release of perforin/serine protease), or via upregulation of cluster of differentiation ligand (CD95L) or TNF-related apoptosis-inducing ligand (TRAIL), induce cancer cell death. For effective use, CTLs need to be trained by tumor dendritic cells (DCs). Type 1 conventional CD103^+^ migrating DCs are antigen-presenting cells (APCs) that elucidate CTLs before cancer cell detection via three different mechanisms: cancer antigen adhered to MHC-I, co-stimulatory molecules (CD80/86 and CD28/152), and pro-inflammatory cytokines (IL-12 and TNF-α) [66]. CTLs and CD4C Th cells develop certain characteristics upon activation that greatly influence the subsequent efficiency of CTL cytotoxic responses [67]. In addition, cytokine-mediated DC licensing activates and supports CD4+ Th cells [67]. APCs also activate CD4^+^ T-cells similarly to CD8^+^ T-cells, except that the tumor antigen epitope is displayed on MHC-II rather than MHC-I. CTLs and CD4C Th cells develop certain characteristics after activation, that greatly influence the subsequent efficiency of CTL cytotoxic responses [68]. CTL phenotypes are commonly defined by the cytokine cocktail that is released via a cytotoxic mechanism to promote cell death. Many studies have demonstrated that CTL-mediated production of IFN-ϒ and TNF-α corresponds to good tumor reduction potential and improved patient endurance [69]. Other investigations have shown that when CD4C Th cells adopt a Th1 phenotype, characterized by the release of IFN-ϒ, TNF-α, and IL-2, patient endurance improves. Although more debatable [70], it has been demonstrated that combining the Th1 response with a Th17 inclination, as defined by IL-17 production, may be even more advantageous. As each T-cell has a unique TCR that recognizes just one antigenic epitope, immunological responses that create a broad measure of antitumor T-cell levels (many T-cell clones) are stronger [68]. The optimum immune response to immunization may vary amongst malignancies. Cancer vaccination can also harness antibody-mediated cytotoxic pathways to limit cancer progression [71] (Figure 3). Antibody-mediated cytotoxicity and antibody-mediated phagocytosis [72] can be used to kill cancer cells when they bind to antibodies. Cancer vaccines based on humoral immunotherapy, aiming to elicit anticancer antibodies in the patient´s body, have mainly used these techniques for passive immunotherapy [73]. Immunological cells responsible for innate immunity (natural killer cells, macrophages, and neutrophils) can identify the attached antibody Fc receptors and drive cell lysis or phagocytosis, once antibodies recognize epitopes on cancer cell surfaces [74]. 

Finally, coactivation of other innate immunity systems, such as T-cells, can help to enhance the adaptive immune response that is sought by cancer vaccinations. The innate lymphoid cells (ILCs), for example, NK cells or invariant NK T-cells (iNKT), offer complementary abilities to CTLs in terms of cancer cell control. To avoid T-cell identification, cancer cells that downregulate MHC-I or overstimulate NK cell-activating receptors (e.g., NKG2D, 4-1BB) can be lysed by NK cells, which have cytotoxic capabilities [75]. When iNKT cells are activated, they secrete cytokines such as Th1 or Th2 in the surroundings and enhance the expression of CD40L. The importance of iNKT cells in influencing adaptive immune responses has been demonstrated by their ability to aggressively boost DC and B-cell maturation, as well as indirectly promoting T-cell responses [76]. Despite this, cancer vaccinations generally fail to target NK or iNKT cells because they do not bear epitopes.

## 4. Cancer Vaccine Adjuvants

An adjuvant boosts or modifies a vaccine’s immunological response. An immunologic adjuvant accelerates, prolongs, or enhances antigen-specific immunogenicity when used with specific vaccination antigens [77]. Antigens alone, in vaccination, are weak adaptogens, and in the lack of an adjuvant, immature DC antigens are not able to elicit a robust immune response. Adjuvants must stimulate immune cells to the injection site, promote cell-mediated antigen transport, and activate antigen-presenting cells (APCs) [78]. Water and oil emulsion adjuvants are commonly used and include montanide ISA-720 and montanide ISA-51, amongst others. Montanide ISA-720 is a 70:30 oil to water blend comprising non-mineral vegetable oil and the surfactant mannide monooleate. Montanide ISA-51 is a blend of monooleate surfactant and mineral vegetable oil that is 50:50 water to oil. These adjuvants trap soluble antigens and inhibit their fast trafficking to lymph nodes by forming a depot system at the injection site [79]. More than 300 clinical trials of cancer vaccines with adjuvants for the treatment of AIDS, malaria, and other diseases have been conducted [3]. 

Adjuvants used in modern vaccines are designed to improve immunity by precisely targeting particular components of the immune system. The more recent adjuvants, such as pathogen-associated molecular pattern molecules (PAMPs), present a threat signal that pattern recognition receptors (PRRs) can decipher and respond to by activating the immune system. Innate cells express many receptors, such as Toll-like receptors (TLRs), nucleotide-binding oligomerization domain receptors, and mannose receptors, on their surface. TLR agonists initiate immunological stimulation and boost vaccination efficacy [80]. It is clear from studies using lymphatic system-targeted TLR agonists that enough inflammatory signals must be provided during immunization to link CD8^+^ T-cell responses with TLR agonist accumulation in draining lymph nodes [81]. A large number of TLR agonists are now being investigated for their potential use as adjuvants in cancer vaccines. TLR agonists that are used the most frequently include polyinosinic–polycytidylic acid with polylysine, carboxymethylcellulose, monophosphoryl lipid A, flagellin, imiquimod, and CPG-ODN [3,82,83,84,85,86]. 

## 5. New Emerging Vaccine Adjuvants

Many unique adjuvants have been identified and used to create cancer vaccines in recent years [87,88,89]. For clinical application, novel adjuvants must overcome difficulties such as unfavorable pharmacodynamics and side effects. Other adjuvants, such as CD40 agonists, which direct the antigen to DC early endosomes and improve cross-presentation, are being investigated to boost the effectiveness of a cancer vaccination. Although CD40 agonist antibodies were not tested as a vaccine adjunct, they have been tested as monotherapy [90]. Several preclinical mouse models demonstrate that a combination of CD40 agonists along with TLR agonists can be used as a vaccination strategy [91]. However, whether their success translates from preclinical to clinical settings is currently undetermined.

Another potential category of cancer vaccine adjuvants is the stimulator of interferon genes protein (STING). STING is a transmembrane protein embedded on the endoplasmic reticulum that upon the response of intracellular DNA, activates type I interferon [92]. Natural and synthetic cyclic di-guanosine monophosphate and dinucleotide derivatives are the main STING agonists that demonstrated antitumor activity in mice [93]. STING activation may induce T-cell apoptosis because T-cells have the highest levels of STING expression, while macrophages and DCs do not exhibit these effects [94]. A STING agonist would need to be supplemented as an adjuvant or along with delivery systems that solely target myeloid cells in murine models to prevent T-cell death in order to be used in a cancer vaccine [95]. To avoid the systemic toxicity that STING agonists might cause, administration at the tumoral site is the recommended method of delivery. Additionally, the differences in how STING agonists attach to murine and human cells have hampered preclinical trials of these compounds [96]. The use of STING agonists in cancer immunotherapies may be constrained by their possible toxicity and lack of precise targeting.

Many cytokines have been proven to act as adjuvants. While studies on immunostimulatory cytokines including IL-2, IL-12 [97,98], IFN [99], and GM-CSF [100] have all been conducted, contemporary research has mostly concentrated on their usage in cellular-based therapeutics and vaccines. Numerous cancer vaccination experiments have included GM-CSF, the most researched immunostimulatory factor [101]. GM-CSF appeared to be a very attractive option in preclinical investigations because it aids in attracting DCs to the injection site, fosters DCs’ maturation, and facilitates antigen presentation, all of which can boost the cancer-specific immune response [102]. GM-CSF has produced poor outcomes in clinical studies, with only a few research studies demonstrating clinical benefits. Most trials have produced variable findings. Preclinical research showed that GM-CSF could grow MDSCs, suppressing antitumor responses [103]. A low dose of GM-CSF also expanded CD14^+^ and low HLA-DR^-^ myeloid cells in clinical trials. In another trial, GM-CSF with incomplete Freund’s adjuvant induced a low T-cell response [104]. Despite these outcomes, several therapeutic trials use GM-CSF as an adjuvant. Inorganic nanomaterials act as immunostimulants, activating and maturing the immune cell system [104,105]. Nanomaterial composition, size, shape, and charge impact adjuvant efficacy. Wang et al. found that TiO2 nanoparticles with nano-spikes stimulated innate immunity. Spiky particles ruptured the cell membrane during phagocytosis by APCs, activating the inflammasome and innate immunity [106]. Spiky TiO2 nanoparticles boosted acquired immunity for cancer immunotherapy when coupled with ovalbumin (OVA) protein [107]. Ferumoxytol, an iron supplement approved by the US Food and Drug Administration (FDA), suppressed breast tumor growth and lung cancer metastases. Jiang and his colleagues also identified manganese as a potential STING pathway regulator [108]. Manganese, being one of the most prevalent metals in mammalian tissues, regulates several functional processes, such as antioxidative tolerance, brain function, and immunological activities [109]. Mn2^+^ raises the sensitivity of cyclic GMP-AMP synthase and STING, which alerts immune cells to infections. Based on these results, Zhang et al. developed a manganese jelly-based colloidal adjuvant (MJ), which has strong adjuvant activity for humoral and cellular immunity with no discernible adverse effects [108]. It was shown that in many antigen models, this colloidal adjuvant increased the antigen display on APCs and induced cytotoxic T-cell responses. Further studies also proved that MJ’s adjuvant exerted its action via NLRP3 and STING activation. MJ-OVA-treated mice produced more OVA-specific antibodies and activated CD8+ T-cells, suggesting that it could be used in cancer immunotherapy [109]. The mevalonate pathway (MVA) causes hypercholesterolemia and bone problems. Interrupting the MVA route with statin and bisphosphonate medications elicited an antibody response [110], demonstrating that it may have vast drug ability for immunotherapy. In animal models, Xia et al. revealed that a lipophilic drug regimen combination of statin and bisphosphonate compounds interacts with three main enzymes involved in the MVA pathway found to be effective adjuvants. In many studies, researchers have also examined the immunostimulant characteristics of various polymers [111]. Gao and co-workers prepared nanocomposites for cancer diagnosis and tailored treatment. Based on proton sponge methodology, they built a series of pH-sensitive polymers (4.0–7.4) [112]. ONM-100, pH-sensitive, polymer-based nanoparticles were developed and licensed for OncoNano Medicine and are currently in a clinical trial phase I study as a surgical imaging agent. The modified nanoparticulate system, PC7A, induced a robust CTL response to eliminate cancer. PC7A nanoparticles triggered STING and boosted immunological responses due to their cyclic 7-element-based side-chain ring. This vaccine formulation increased cytosolic bioavailability and cross-presentation and enhanced CD8+ T-cells [113,114]. 

## 6. Nanocarrier Systems as Cancer Adjuvants

Liposomes were the first commercially available particle drug delivery technology for antigen administration. Safety and adaptability are key to liposomal vaccination clinical translation and research success. Liposomal pharmaceuticals such as DoxiI^®^ and AmBisome^®^, as well as vaccine preparations such as Epaxal^®^ and Inflexal^®^, illustrate liposomes’ safety and tolerability [115]. Eiji et al. prepared surface-engineered, pH-sensitive fusogenic liposomes of DOPE/egg yolk phosphatidylcholine and linear and hyperbranched poly(glycidol) derivatives. These surface-engineered liposomes may easily transfer fluorescent-labeled antigen into DC cytoplasm, prompting DC maturation and, through MHC-I-mediated antigen presentation, decreasing tumor burden [116]. Liposomal administration of CpG ODNs elicited substantial antitumor effects in an animal model of neuroblastoma, whereas CpG alone failed [117]. When utilized to deliver DNA or RNA complexes to mice, liposomes increased APC absorption and stimulation, resulting in anticancer effectiveness [118]. Recently, a novel lipopolyplex vector for mRNA delivery was developed by integrating a multivalent cationic lipid and the immune adjuvant α-galactosylceramide to target dendritic cells (DCs) [119]. 

Virosomes, another type of liposome, are spherical structures into which phospholipids from the virus envelope and viral spike proteins have been integrated. Although they were discovered in 1975, the first human virosomal vaccine, Inflexal V for influenza, was launched in 2009 [120]. For the prevention of malignancies caused by human papillomavirus types 16 and 18 (CervarixTM and Gardasil^®^), vaccinations based on virions are currently on the market [121,122]. The ability of virosomes to transport tumor-targeted antigens to the APCs and elicit an immune response without multiplying or becoming infectious made them a popular choice for use in cancer vaccines [123]. As a result, virosomes boost antibody and T-cell responses [124,125], as seen in a phase I clinical trial of patients with metastatic breast cancer [126,127]. An electromagnetic substance embedded in HA-virosomes in the presence of an externally applied magnetic field has been considered a revolutionary scaffold in the targeting of brain tumors [128]. 

ISCOMs were first invented by Morein in 1982 and mainly contained the Quil A saponin adjuvant extracted from the bark of the *Quillaia saponaria* Molina tree, along with cholesterol and phospholipids [129]. In the ISCOMs, cholesterol and saponins interact firmly to form complex cage structures of 40 nm in size, in which proteins and adjuvants are entangled. This cage-like structure provides stability and reduces the hemolytic potential of saponins [77]. Pabreja et al. prepared an ISCOM-based tuberculosis vaccine loaded with antigen 85. Immune study results showed that BALB/c mice immunized with ISCOMs have high levels of IgG_1_ and showed significant importance in the development of humoral and cellular immune responses after pulmonary immunization [130]. Cibulski and coworkers prepared ISCOMs with saponin. They explored the immune potential of ISCOMs formulated with a saponin derived from *Quillaja brasiliensis* with OVA antigen, phospholipids, and cholesterol. Immunological study results showed that these formulations elicit robust humoral responses (IgG1 and IgG2) and tumoral responses (T-cell proliferation and Th1 cytokines) after subcutaneous administration. Its intranasal delivery induces serum IgG, IgG1 level, and mucosal IgA [131]. The only problem associated with using ISCOMs in vaccine formulation is their toxicity profile, which further induces hemolysis [132]. 

Another vaccine adjuvant carrier system are lipid nanoparticles (LNPs) with biodegradable characteristics [133]. Xu et al. prepared calcium phosphate LNPs for the Trp2 peptide vaccine delivery system in melanoma treatment. The addition of phosphorus-serine residues at the N-terminal of the peptide increases the calcium phosphate co-precipitation. Mannose-modified LNPs encased CpG ODN. Vaccination with LNPs induced a significant CTL immune response, inhibiting tumor growth in B16F10 subcutaneous and lung metastatic models [134]. Polymeric NP (PNP) adjuvants protect/stabilize vaccination antigens until they achieve the target location. PNPs can be made from a variety of polymers, including FDA-approved polymers such as PLGA, PLA, PACA, polyanhydrides, and chitosan in sustained drug delivery [78,135]. Liu et al. proved the antigen stability, antigenicity, and release kinetics of MUC4β and anhydride monomer 8-bis(p-carboxyphenoxy)-3,6-dioxaoctane (CPTEG) and 1,3-bis(p-carboxyphenoxy)hexane (CPH) nano-vaccine in a 20:80 ratio [115]. In vitro investigations indicated continuous MUC4 protein release without protein degradation or epitope loss. The synergistic activity of MUC4 and CTPEG (20):CPH (80) nanoparticles elicited an MUC4-specific IgG immune response [136]. 

Inorganic particles are used to target tumor-associated antigens to solid tumors. Gold nanoparticles (GNPs) are safe and inert and can be manufactured with well-regulated dimensions and forms [137,138]. Due to their easy synthesis and tunable size, Kang evaluated the influence of OVA-carrying GNPs with dimensions of 10, 22, and 33 nm on delivery to draining lymph nodes (LNs) and CD8+ T-cell immune responses. Results revealed that delivery to LNs and CD8+ T-cell responses’ induction was higher with 22 and 33 nm OVA-GNPs [139]. Zhang et al. prepared multilayer polyelectrolytic GNPs with anionic poly I:C and antigenic peptides using a solvent-free technique, via which components are self-assembled to form multilayers and which further induced higher antigen-specific CD8+ T-cells [140]. 

Aluminum-based adjuvants are used a lot and have been approved by the FDA for human use. In an aqueous solution, the traditional aluminum hydroxide breaks down into particles of 1–20 m in size and weakly boosts immune responses. Li and his colleagues synthesized aluminum hydroxide NPs (112 nm) loaded with OVA and *Bacillus anthracis* protective antigen in their study. The result concluded that these NPs, as compared to microparticles, induce a more robust immune response and less inflammation at the site of injection. This can be an efficient and safer alternative for adjuvant vaccine delivery [141]. Other types of inorganic NPs, such as Fe, ZnO, SiO_2_, and TiO_2_, are promising antigen/adjuvant delivery vehicles. The primary concern associated with inorganic NPs is that they may be deposited in various tissues and organs and induce prolonged toxicity [142,143,144]. 

## 7. Peptide-Based Cancer Vaccines

**Figure 4:**
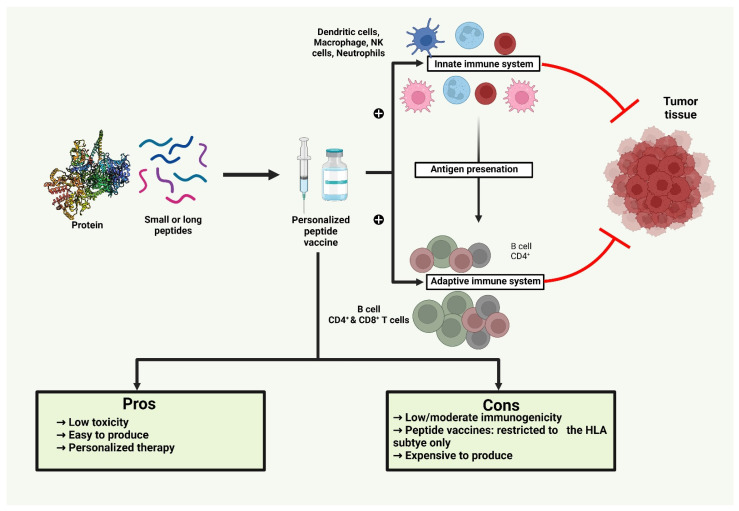
Representation of activation of CD4^+^ and CD8^+^ epitopes through antigen presentation regulated by the innate immune system, which inhibits proliferation of tumor tissue (Created with biorender.com).

The use of single agents or combinations of proteins, heat-shock proteins (HSPs), anti-idiotype antibodies, fusion proteins, peptides, and agonists to stimulate a particular immune response against cancer has been extensively investigated [145,146,147,148,149]. Additionally, utilizing synthetic peptide vaccination techniques, customized personalized vaccines based on antigens specific to particular tumors can be created. Typically, these cancer vaccines are composed of 20–30 amino acids with particular epitopes from antigens that are anticipated to elicit a potent immune response [150]. In comparison to inactivated cancer cell vaccines, peptide-based vaccinations (Table 4) induce a more focused immune response, targeting significant neutralization epitopes. This immunological advantage of such vaccines is referred to as immunodominance [151]. In general, peptide-based cancer vaccines require both CD8^+^ and CD4^+^ T-cell epitopes (Figure 4). CD8^+^ T-cell epitopes activate CTLs via the antigen cross-presentation technique, whereas CD4^+^ T-cells stimulate helper T-cells to maintain CTL activity [152]. However, these vaccinations can only partially lead to the activation of CD4^+^ and CD8^+^ subsets [153]. 

The length of the peptide chain has a significant impact on peptide-based cancer vaccines’ effectiveness. Short peptides—those with 8 to 12 amino acids—that are produced chemically often have a short half-life in vivo and are rapidly broken down in blood. These vaccinations often interact with nucleated cells’ interfaces of human leukocyte antigen-1 (HLA-1) without affecting APCs. Due to their small size, these peptides do not allow for the diversity required for the general population to have a significant level of HLA polymorphism, and hence it has been discovered that they are solely HLA-type specific. However, without concomitant activation of CD4^+^ helper T-cells, this could result in tolerance or momentary induction of CD8^+^ T-cells and momentary activation of CTLs [154,155,156]. Longer peptides, typically 20 amino acids, are utilized in peptide vaccines, and these vaccines are more likely to have both longer CD4^+^ T-cell epitopes and shorter CD8^+^ T-cell epitopes, and hence tend to trigger both CD4^+^ and CD8^+^ T-cell activation. Since they are occupied and processed by APCs before being loaded onto MHC, these vaccines are more immunologically effective, long acting, and stable than shorter peptides. As a result, B-type lymphocytes produce antibodies that are extremely efficient and long-lasting against tumors [157,158]. 

Peptide-based vaccines frequently had multiple epitopes against various targets in clinical trials, such as NCT02362451 and NCT02362464, as opposed to in vitro studies, which typically focused on a single epitope and have shown to be more effective because these are well-tolerated and have a variety of clinical benefits against various cancers. Thus, a good treatment system against cancer is provided by a peptide created from several peptides [159,160]. Derouazi, in 2015, created a unique class of cancer vaccines based on recombinant proteins, by combining recombinant protein with Z12, which provides a variety of CD8^+^ and CD4^+^ T-cell epitopes expressed by MHC class I and class II alleles. It facilitates the loading of proteins into the antigen-processing machinery of dendritic cells and has demonstrated prolonged life in an orthotopic model of aggressive brain cancer [161]. The lengthy synthetic peptide-based cancer vaccine (SVX vaccine) targeting surviving TAA was created and studied by Onodi et al. It has three distinct peptides that represent CD4^+^ and CD8^+^ epitopes that bind to HLA-1 and HLA-2 components. In mice engrafted with colorectal cancer and B lymphoma, this vaccination displayed a tumor growth reduction, and this effect was correlated with the emergence of surviving specific T-cell responses [162]. 

Personal neoantigen vaccine, a novel peptide-based cancer vaccine, has been demonstrated to be safe, efficient, and capable of inducing robust T-cell responses. Such vaccines are administered to APC with the goal of stimulating an individual’s immune system to identify and eradicate cancer cells by exposing T-lymphocytes to tumor-specific neoantigen. Cold tumors can become “hot” cancers when T-cells with tumor antigen-specificity are incorporated into the tumor microenvironment (TME), resulting in a greater anticancer response. The iNeo-Vac-P01 vaccine can increase pancreatic cancer’s currently low clinical efficacy, according to a phase I trial performed on 20 patients with advanced pancreatic cancer and low tumor mutation burden (TMB) [163]. This customized vaccination has demonstrated a striking rise in antigen-specific TCR clones, CD4^+^ or CD8^+^ effector memory T-cells, and greater peripheral IFN-γ titer levels, indicating the potential of such a customized vaccine to activate T-cells and lead them to target malignant cells.

Peptide-based vaccines have several benefits, but there are also a lot of obstacles to consider while developing them. Genomic changes of neoantigens, such as deletions and mutations, may trigger endogenous T-cell immune responses in a variety of tumor types, even if certain severe side effects may be connected to genetic abnormalities in tumors with high-affinity T-cell receptors [164,165]. The ideal TAA should also be widely expressed in a variety of tumor types and play a crucial role in oncogenic processes or cancer cell survival to minimize immune escape by mutations or antigen loss by tumor cells. Therefore, two widely used techniques exist for discovering novel TAAs: direct immunology, which begins with patient-derived autologous tumor-specific CTL clones specific for an undiscovered epitope, and reverse immunology, which starts with a predicted epitope. Even while peptides recognized by helper T-cells on MHC-II molecules may increase efficacy, it is highly challenging to predict the immunogenicity of MHC-II-restricted peptides since they are more complex than MHC-I. Bioinformatic tools and algorithm prediction programs are frequently used nowadays to define peptides that can bind MHC-I or MHC-II molecules [166]. Moreover, peptides connected to MHC molecules on the cell surface can be found via mass spectrometry analysis. The most suitable candidate peptides for a vaccine can be chosen by combining information from gene expression data, epitope predicting algorithms, and mass spectrometry analysis [3,167]. 

### Role of Adjuvants in Improving the Efficacy of Peptide-Based Cancer Vaccines

Immune responses are not sufficiently stimulated in vivo by peptide vaccines administered alone. As a result, strong immune-stimulators or strong adjuvants are required for the delivery of peptide-based cancer vaccines. Adjuvants and antigens must be administered together in order to stimulate the immune system effectively while preventing autoimmunity or toxicity [165]. The adjuvant and antigen may be delivered through a delivery technique, as already discussed, or they may be combined to improve targeting. PAMPs and pro-inflammatory cytokines are currently used as adjuvants to broadcast a danger signal that pattern recognition receptors (PRRs) will recognize and react to by inducing an immune response. TLR agonists are strong adjuvants that mimic microbial stimulations and have been shown to enhance epitope-induced CTL memory activation and vaccination efficacy in malignancies [168]. Additionally, peptide-based cancer vaccines have been utilized in conjunction with Montanide ISA^™^51 VG (ISA 51) as an adjuvant. It was shown that Montanide ISA^™^ 51 induced CD4^+^ and CD8^+^ T-cell responses in individuals who received vaccinations with long peptides derived from the oncoproteins E6 and E7 [79]. By improving APCs’ capacity to present tumor antigen peptides on their surfaces via MHC-I for CD8^+^ T-cells to recognize, heat-shock protein (HSP) fused to cancer vaccines may enhance the antitumor immune response [169,170]. The STING protein agonist, a transmembrane protein that incites a potent type-I IFN response, belongs to another family of novel emerging adjuvants. According to Rossi et al., the combination of a peptide-based cancer vaccine with STING treatment enhanced the therapeutic effects of immunization, resulting in prolonged control and slower growth of B16-OVA and TC-1 tumors in mice [171]. Clinical trials have indicated the potential of cytokines, including interleukin-2, granulocyte-macrophage colony-stimulating factor (GM-CSF), and IFN-γ, as adjuvants for cancer vaccines. GM-CSF has been used as a vaccine adjuvant in antitumor immunotherapy for prostate cancer, adenocarcinoma, breast cancer, and lung cancer in clinical trials such as NCT00841399, NCT03579654, and NCT00028496 [172]. 

## 8. Virus-Based Cancer Vaccines

An oncolytic virus is a novel approach that specifically ends up killing tumor cells while also stimulating antitumor responses [173]. Tumor cells infected with the oncolytic virus generate free radicals as well as cytokines that also stimulate immune cells, which tends to result in oncolysis and causes the release of chemical compounds such as TAAs. Vectors can powerfully stimulate adaptive immune responses because the immune system interprets them as invading particles, which can have foreseeable as well as durable immunologic consequences. Besides this, a wide range of recombinant viruses have also been shown to infect and to articulate their transgenes on APCs, including dendritic cells. Due to the perceived immune system’s enhanced ability to detect TAAs, the percentage and the effectiveness of cytotoxic T lymphocytes that invades the cancerous cells and further, conveys the tumor antigen(s) which are encrypted in the immunization vector, seems to have increased [174]. Repressed or replication-defective viral vectors are recommendable from a safety aspect, and they constitute a major part of cancer vaccines based on viruses [3]. Moreover, the potency of inactive entire viral (Adenovirus) vaccines for COVID-19 or Ebola treatment has been promising [175,176]. Nevertheless, there is a negative aspect in using these viral vaccines. Due to the ability of viral vectors to elicit immune function, they can activate the vector as well as future immunizations. Hepatitis virus C, HBV, Alphaviruses, Adenoviruses, and viruses from the Orthopoxvirus genera, as well as the extracts of Vaccinia virus, and even the members of the Avipoxvirus genera, such as the Fowlpox and Canarypox, are perhaps the most prevalent viruses used for the development and progression of cancer vaccines, such as the ALVAC-CEA vaccine [177,178,179]. Poxvirus vectors are large-enveloped, double-stranded DNA (dsDNA) viruses that belong to the Poxvirus family and have robust recombination and replication efficiency and can proliferate in the cytoplasm of a variety of hosts (vertebrate and invertebrate species) and tumor cells. The tumor antigens can be mediated by both class I and class II MHC channels through internalized transgene expression that triggers CD4+ and CD8+ T-cells [180]. 

Adenoviruses are frequently utilized as vectors for transducing certain genes. Oncolytic as well as non-replicating Adenovirus-based vaccines have apparently demonstrated potential in preclinical and clinical studies (NCT00583752, NCT02285816). Adeno-associated virus vectors are reliable and proliferate in experimental settings [181]. This allows for simple vector design by biologists, involving reconfiguring of the virus’ tropism to enhance transduction of target cells and DCs that are deficient in the crucial adeno-receptor (Ad receptor). Adenovirus infections can lead hosts to produce antibodies that are neutralizing, and further minimize the need for vaccination shots [182,183]. Additionally, experimental clinical studies such as NCT00108732 indicate that repeated booster doses of a Fowlpox vaccine may not induce host anti-vector immune functions. In parallel to adenovirus, the cancer vaccination infrastructure also employs lentivirus and Vaccinia virus as vectors [184,185]. Comparable to Adenovirus, Lentivirus as well as Adeno-associated virus have the peculiar ability to articulate the transgene in non-dividing cells over an extended period of time. The bird virus, known as Avipox (Avian poxviruses, genus Avipoxvirus), replicates in mammalian cells. Both vectors effectively infect APCs without incorporating into DNA, causing potent immune reactions. The vaccinia virus was repeatedly passed through the fibroblasts of chick embryos to produce the substantially attenuated strain of Vaccinia, termed as modified Vaccinia Ankara (MVA). Following infection, MVA is still able to regenerate DNA in mammalian cells, but it is no longer able to produce infectious viral particles [186]. 

### 8.1. Prostate-Specific Antigen plus a Triad of Co-Stimulatory Molecules

The PSA-TRICOM vaccine framework employs both recombinant vaccinia virus (RV) as well as recombinant Avipox (Fowlpox, rF) for vaccination. Every PSA vaccination incorporates three immunological co-stimulatory compounds known as TRICOM (CD80, ICAM1, and LFA-3), an agonist epitope, as well as PSA transgenes [187]. In a phase II clinical trial, 125 men with metastatic, castration-resistant prostate cancer were administered 6 shots with a Fowlpox virus-encoded PSA after receiving a prescribed dose of Vaccinia virus-encoded PSA and GM-CSF (PROSTVAC-VF). The findings of this phase II trial disclosed a 10-month overall improvement in survival rate, when compared to a control group carrying an empty vector [188]. Regrettably, a massive phase III study did not reproduce these outcomes, leading to the trial’s termination [189]. Even though specific T-cells have been stimulated, the immunomodulatory tumor microenvironment may have played a negative role in the modulation of the immune response against the tumor. PANVAC-VF, another vaccine based on poxviruses, consisted mainly of a primer shot encoding CEA(6D), TRICOM, and MUC1(L93), accompanied by booster shots that encode the same transgenes in rF. MUC1(L93) and CEA(6D) have a specific amino acid altered to enhance the immunogenicity, and this represents carcinoembryonic antigen and mucin 1 glycoprotein, respectively [189]. In the metastatic cancer pilot study, 25 people underwent 135 cycles of PANVAC. Only 7% of PANVAC cycles were found to be related with grade 2 flu-like symptoms, and a grade 3 transient syncope that occurred during a flu-like illness was possibly attributable to PANVAC [189]. 

### 8.2. Strategies for Optimization of Virus-Based Cancer Vaccines

TME enhancement is an approach to maximize the efficiency of cancer vaccines based on viruses. As our mechanistic understanding behind the immunosuppressive nature in TME has evolved, numerous strategies incorporating viral vaccinations are becoming feasible. It was discovered that Treg, a vital immunosuppressive cell, is controlled by the protein YAP, a major coactivator of the Hippo pathway. A YAP insufficiency would result in Treg dysfunction. As a result, interfering with the Treg-mediated immunosuppressive TME and further inactivating YAP could improve the antitumor efficacy of the viral vaccine [190]. As it has been demonstrated that TGF can elicit immunological suppression by affecting a range of immune cells, such as T-cells or NK cells [191], another approach is to combine TGF with a proven viral vaccine. Additionally, PD-L1 inhibitors and vaccines based on viruses have been used together and evaluated. The integration of the viral vaccine and PD-L1 inhibition leads to long-term tumor-free survival in a tumor model. M7824, a novel bifunctional anti-PD-L1 and TGF-β, has been reported to help virus-based cancer vaccines work together more effectively [192]. In relation to this standard treatment, virus-based vaccines with immune-regulating components are being developed to obstruct the TME. For example, an oncolytic viral vaccine such as BT-001 can express anti-CTLA4 antibody as well as GM-CSF [193]. 

## 9. Nucleic Acid-Based Vaccines

**Figure 5:**
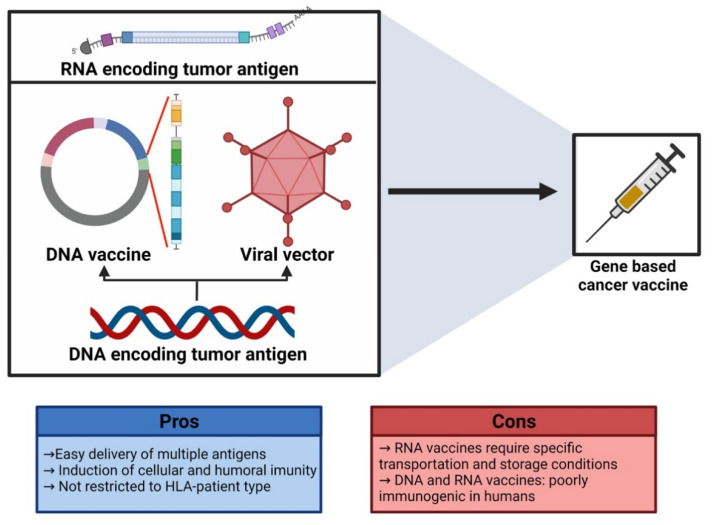
Diagrammatical representation of gene-based cancer vaccines (created with biorender.com).

When therapeutic uses are considered, nucleic acid vaccines offer good potential in the fight against infectious illnesses as well as cancer. Nucleic acid vaccines differ from conventional vaccinations because they are very effective and inexpensive. As a result, nucleic acid vaccines may be beneficial for both the diagnosis as well as for the treatment of diseases. However, nucleic acid vaccines’ limited immunogenicity and stability have hindered their development. As a result, numerous studies have been performed to enhance their immunogenicity as well as stability through enhanced delivery systems, thereby enabling advancements for medical applications [10]. Additionally, the targeted antigen in the pathogen is the only part of the immune response that is triggered by nucleic acid vaccines. Vaccines made from nucleic acids (Figure 5), such as DNA as well as RNA, demonstrate great potential in the medical field [194]. 

### 9.1. DNA Vaccines

DNA vaccines are an intriguing approach in the immunotherapy of cancer. These vaccines were discovered in the 1990s as a result of DNA-mediated influenza vaccination [195]. DNA vaccines consist of bacterial plasmids with double-stranded DNA that have two replication origins. They also have a polyadenylation motif and a regulator that resembles the human cytomegalovirus. The bovine growth hormone (BGH) gene provides the polyadenylation sequence, and the CMV promoter causes the transgene to be overexpressed in the host cell of the mammals. DNA vaccines are referred to as vaccines of the third generation. These vaccinations constitute of genetically implanted DNA which encodes for an antigen of intended bacteria or viruses via a robust promoter present in the DNA plasmids. The antigens that are encoded would then elicit a strong immune function in the host [196]. DNA vaccines must reach the nucleus to undergo transcription and then the encoded antigens will be translated inside the cytoplasm. MHC-I and MHC-II molecules then further process the antigen and convey it to CD8^+^ T- and CD4^+^ T-cells to initiate immunogenicity [197]. The Food and Drug Administration of the USA regulates the DNA vaccines for medicinal and therapeutic uses in the veterinary field. Immunoregulatory cognition has been found to be improved by this vaccine [198]. The channel via which DNA vaccines are administered is thought to have a significant impact on the effectiveness of the vaccine. Targeting distinct APCs inside diverse tissues can help a vaccine to induce the necessary response [199]. These vaccines are frequently administered intravenously as well as topically [200]. Parenteral routes, such as intravenous, subcutaneous, and intramuscular, are most frequently used for the delivery of DNA vaccines. These routes activate tissue-specific APCs, which in turn stimulates cellular and humoral immune reactions. Due to its ability to induce localized immunity at body regions that serve as the characteristic entry points for numerous microorganisms, the delivery of DNA vaccines via mucosal channels has recently attracted a lot of attention [201]. DNA vaccine action mechanisms are classified into three types: The first pathway includes the entry of the DNA inside the somatic cell, for example the muscle cell, which undergoes translation, and after that MHC-1 molecules transmit DNA-encoded antigens directly to cytotoxic CD8^+^T-cells [202]. In a few instances, the antigen expressed by DNA in somatic cells is liberated by emitting the apoptotic bodies, and this would include the second pathway. Such peptides are then endocytosed by phagocytic cells, metabolized by APCs, and cross-presented to CD4^+^ T-cells by MHC-II molecules. The direct DNA transfection of APCs includes the third route [200]. Such vaccines have significant advantages that include convenience of design, relatively low cost, long-term stability, an optimum dissolution rate, and even the capacity to be speedily amended [3]. Despite the poor immunogenicity of DNA vaccines in clinical studies, technological advancements such as delivery systems and the use of immuno-stimulatory agents such as adjuvants may contribute to a better result [198]. The concept of using xenogeneic antigens in DNA vaccines to cure melanoma and overcome systemic immune tolerance has recently been studied. In recent cancer studies, xenogeneic p53 antigen is employed to produce DNA vaccines to combat colon cancer [203]. Furthermore, many new preclinical studies have shown that a DNA vaccine encoding the prostate-associated antigen prostatic acid phosphatase (PAP) can elicit PAP-specific CD8+ T-cell immune responses, as seen in a phase I/II study employing a DNA vaccine that encoded human PAP conducted in 22 prostate cancer patients [204]. There are many methodologies for increasing the therapeutic efficacy of DNA vaccines. A potent promoter sequence is required for efficient transcription. Powerful promoters have also been shown to enhance the expression of antigen efficiency [205]. Enhancing the layout of tumor-specific antigens is also critical in enhancing the DNA vaccines. Immuno-stimulatory cytokines can also aid in enhancing the vaccine’s impact on effector T-cells. They are typically encoded by the vaccine that incorporates antigens, by some other plasmid, and are administered as proteins along with the vaccine. The most frequently employed cytokines in the latest research include IL-12, GM-CSF, as well as IL-2. Various approaches are combined with DNA vaccinations to increase their efficacy, including endocrine therapy and radiotherapy [206]. 

### 9.2. RNA Vaccines

RNA vaccines usually consist of a DNA template strand that encodes the intended antigen(s) and messenger RNA produced through in vitro transcription by using bacteriophage RNA polymerase. The assigned mRNA transcripts are directly translated into the cytoplasm by the host cells after they have ingested them, and thereafter the generated antigens are subsequently delivered to APCs to trigger an immune response. As an alternative, dendritic cells could be given to the host combined with either tumor-related antigen mRNA or entire tumor RNA to trigger a specified immune system response [207]. These vaccines are favored as they have almost similar positive traits, as exhibited by the DNA vaccines, and in addition, provide some further advantages. Unlike DNA, RNA can transfect a cell by simply entering the cytoplasm, where translation occurs. RNA vaccines can often be extracted from a tumor sample and be further amplified, using methods such as polymerase chain reaction (PCR). This process produces immense quantities of patient-specific antigens [208,209]. By transmitting co-stimulatory signals, such as those through the toll-like receptors TLR3, TLR7, and TLR8, RNA can function as an adjuvant. These factors have led to increased significance to the development and creation of RNA vaccines. Trans-replicating, self-replicating, as well as non-replicating RNAs constitute the three primary types of mRNA vaccine structures. Numerous cancer vaccine approaches have relied either on self-replicating or non-replicating RNAs. Non-replicating mRNA encodes the specific antigen that is promptly translated into protein after entering the cytoplasm of the target cell. This apparently produces protein-rich expression that progressively declines with time [210]. Through in vitro transcription or the extraction of the whole transcripts, the development of non-replicating mRNA is rather straightforward. In contrast, the transgene of interest is also included in self-replicating constructions, together with the viral RNA-dependent RNA polymerase (RdRp), which replicates the viral genome [211]. Trans-replicating, or splitzicon, RNAs transport the desired transgene and the viral RdRp on distinct transcripts to minimize the amount of RNA vectors that must be enclosed during manufacture. A crucial component in generating mRNA vaccines for cancer therapy is the optimal correlation of intrinsic sensing of the mRNA structures, along with the production of vectors [212,213]. Initial mRNA-based therapies have demonstrated potential to treat cancer. Clinically, these substances have the potential to alter the immunosuppressive TME and act as strong T-cell stimulants. Furthermore, more innovative technologies are required to create effective RNA-based cancer therapeutics due to the intrinsic variability of cancers, both within and between clinical indications. To gain a therapeutic edge, ongoing chemical engineering projects are modifying mRNA vectors to adapt an optimum balance between antigen expression and innate immune detection. To combat systemic or local tolerance, the tumor antigen target type may necessitate various intensities of immunostimulatory signaling [214]. The most promising implementation of mRNA vaccines is to vaccinate cancer patients with autologous dendritic cells (DCs) infused with mRNAs processing tumor-associated antigens (TAAs). Many of these efficacious mRNA vaccines are developed for brain cancer, ovarian cancer, as well as for prostate cancer in the respective clinical trials NCT02808416, NCT01334047, and NCT01446731 [215]. A phase I clinical trial for the treatment of metastatic melanoma is being conducted with BNT111, a product of BioNTech SE. This vaccine targets the tumor-associated antigens that are present predominantly in melanoma, such as MAGEA3, tyrosinase, TPTE, and NY-ESO-1. Pursuant to this, tumor cells should be eradicated by the immune system as a consequence [216]. 

## 10. Conclusions and Future Perspectives

Cancer vaccines and immunotherapy rely on a thorough understanding of tumor immune evasion mechanisms. Recent studies have examined the tumor microenvironment and drawn conclusions about internal and external resistance influencing the therapeutic response at distinct stages of the disease. Prior successful and clinically impactful vaccination attempts may influence future therapeutic platform design. Antigen prediction findings and innovative adjuvant systems have increased vaccination personalization. Antigens may be administered as peptides, mRNA, or DNA vaccines or displayed on DC xenografts. Different vaccination platforms, immunomodulation, and potential cancer therapeutics (chemotherapy and radiation) must be combined to fight tumor resistance. The timing, sequence, and dose of each component must be carefully determined for effectiveness. Preclinical and clinical studies show that cancer immunotherapy vaccines can destroy target cells in a variety of tumor types with little side effects by activating T lymphocytes against tumor antigens. Reduced immune evasion and successful tumor eradication may be achieved using vaccines that target numerous neoantigens. To increase antitumor immunity, cancer vaccines present an appealing way to combine with currently used immunotherapeutic approaches, such as immune checkpoint inhibitors, oncolytic viruses, and other immunomodulators. Such a combinatorial therapeutic approach can compensate for the limitations of each therapy when used alone. To activate, expand, and facilitate the anticancer immune response, desirable multiplicative and/or synergistic therapeutic medications with cancer vaccines should counteract the limited immunosuppression mechanisms responsible for immune escape. To further optimize and design the therapeutic cancer vaccines, some important considerations should be followed during preclinical and clinical translational efforts, such as a robust vaccine design that quickly detects the most favorable combination approaches as per the tumor environment for an optimal immune response. Therefore, cancer immunotherapies are promising medications for eliminating tumors and creating immune surveillance. Much effort is required to identify neoantigens, develop combination therapy, and optimize vaccination technologies until cancer vaccines become a viable therapy.

## Figures and Tables

**Table 1:** Generally employed categories for cancer vaccines.

Type of Vaccine	Method for Implement	Significance	Reference
Dendritic cell vaccine	Immune cell stimulants are used to develop a significant number of dendritic cells (DCs) from the isolated dendritic cells from patients.	T-cells’ reprogramming	[16]
Antigen vaccines	Those antigens are administered intravenously to patients with cancer, stimulating the immunological system to create more antibodies or cytotoxic T-cells.	Enhance human T-cell reactivity against tumor	[17]
Anti-idiotype vaccine	Triggering an immune response.	Primary target lymphoma	[18]
DNA vaccine	DNA from the patient’s cell is administered to other cells, instructing them to continually generate specific antigens.	Causes an immune response by increasing the production of T-cells.	[19]
Tumor cell vaccine	One of the earliest tumor vaccines to be used, autologous and allogenic tumor cells.	The immune system needs every relevant tumor antigen to produce a successful anticancer reaction. Additionally, it allows the creation of cancer vaccines without the knowledge of precise antigen(s)/neoantigen(s)	[20]

**Table 3:** Immunotherapies for cancer currently on the market [60].

Paradigm	Example	Approved *	Target
mAbs that target tumors	Herceptin	Yes	Herceptin is approved for the treatment of early-stage breast cancer that is human epidermal growth factor receptor 2-positive (HER2+).
Transfer of adoptive cells	Vemurafenib	No	Vemurafenib is used to slow the growth of certain types of cancer cells.
Oncolytic viruses	RIGVIR, Oncorine, and T-VEC	Yes	The treatment, which is injected into tumors, was engineered to produce a protein that stimulates the production of immune cells in the body and to reduce the risk of causing herpes.
DC-based therapies	_	No	Targeted treatment that involves extracting and manipulating components of a patient’s immune system (the dendritic cells) to boost its chances of eliminating unnoticed cancer cells.
Vaccinations based on peptides	TAS0314	Yes	Dramatically suppressed tumor growth.
Immunomodulatory mAbs	Rituximab (Rituxan)	Yes	It specifically targets the CD20 protein. B-cells, a type of white blood cell, have CD20, and it is indicated in patients for non-Hodgkin’s lymphoma and chronic lymphocytic leukemia.
Immunostimulatory cytokines	IFN-α	Yes	Approved for the treatment of some hematological malignancies and AIDS-related Kaposi sarcoma.
Immunosuppressive metabolism inhibitors	Rapamycin	No	Decrease the risk of organ transplant patients to develop cancer.
PRR agonists	Imiquimod	Yes	To achieve the purpose of regulating immunity and treating tumors.
ICD inducers	Radiation	Yes	The best-characterized inducer of immunogenic cell death.

* In one of its forms for use in cancer patients, by the US Food and Drug Administration or an equivalent regulatory agency worldwide.

**Table 4:** Investigational trials examining the efficacy of TAAs or peptides in treating cancer patients.

Cancer Type	Trial Phase	Action	TAAs	Notes	Trial No.
Bladder carcinoma	I	Completed	PPV	Atezolizumab with Hiltonol^®^ adjuvanted intervention	NCT03359239
Brain tumor	I	Recruiting	Multiple	Varlilumab with Hiltonol^®^ adjuvanted intervention	NCT02924038
Breast carcinoma	I/II	Completed	FOLR1	Combined with cyclophosphamide and GM-CSF	NCT02593227
Breast carcinoma	II	Active	FOLR1	Combining GM-CSF-adjuvanted therapy with cyclophosphamide	NCT03012100
Breast carcinoma	II	Recruiting	HER2	Combined with GM-CSF	NCT02636582
Breast carcinoma	I	Active	Multiple	Durvalumab in combination with the adjuvanted interventions of montanide, ISA-51, and Hiltonol^®^	NCT02826434
Breast carcinoma	I	Active	Multiple	Added to pembrolizumab	NCT03362060
Breast carcinoma Gastric carcinoma	I	Completed	HER2	Intervention with GM-CSF, imiquimod, and cyclophosphamide	NCT02276300
CRC	I	Completed	Multiple	Chemotherapy in addition to a montanide ISA-51- adjuvanted intervention	NCT03391232
Glioblastoma	I/II	Completed	WT1	Only one adjudicated agent	NCT02750891
Glioblastoma	II	Recruiting	WT1	Added to bevacizumab	NCT03149003
Glioma	I	Completed	IDH1	ISA-51 adjuvanted with montanide	NCT02454634
Glioma	I	Recruiting	H3	Adjuvanted with montanide ISA-51 and Hiltonol^®^	NCT02960230
Glioma	II	Recruiting	n.a.	In conjunction with Hiltonol^®^	NCT02358187
HCC	I/II	Completed	Multiple	Combined with cyclophosphamide and CV8102- adjuvant intervention	NCT03203005
HPV tumor	I	Completed	p16	ISA-51 adjuvanted with montanide	NCT02526316
Kidney cancer	I	Recruiting	PPV	Ipilimumab with Hiltonol^®^ adjuvanted intervention	NCT02950766
Kidney cancer	I/II	Unknown	Multiple	Montanide ISA-51 and GM-CSF were added	NCT02429440
Leukemia	I	Recruiting	PPV	Intervention with Hiltonol^®^ adjuvant and cyclophosphamide	NCT03219450
Leukemia	I	Unknown	Multiple	Montanide ISA-51 and GM-CSF were added as adjuvants	NCT02240537
Leukemia	II	Recruiting	PPV	Lenalidomide with imiquimod adjuvant	NCT02802943
Lung cancer	I	Recruiting	PPV	Intervention with Hiltonol^®^ adjuvant and pembrolizumab, cisplatin as well as pemetrexed	NCT03380871
MDS	I/II	Completed	WT1	Only one adjudicated agent	NCT02436252
Melanoma	n.a.	Completed	MART-1	Only one adjuvanted agent	NCT02320305
Melanoma	I	Completed	Multiple	Combined with GM-CSF	NCT02696356
Melanoma	I/II	Recruiting	Multiple	When used with trametinib and dabrafenib	NCT02382549
Melanoma	I/II	Terminated	Multiple	Ipilimumab and montanide ISA-51 adjuvanted intervention	NCT02385669
Melanoma	I/II	Terminated	Multiple	Combining cyclophosphamide with the adjuvanted interventions of montanide ISA-51 and Hiltonol^®^	NCT02425306
Melanoma	I/II	Completed	Multiple	Added to pembrolizumab	NCT02515227
Melanoma	I/II	Recruiting	IDO1 and PD-L1	Nivolumab in addition to a montanide ISA-51- adjuvanted intervention	NCT03047928
Melanoma	II	Completed	NY-ESO-1 and MART-1	Combining DC vaccination with a montanide ISA-51 and Hiltonol^®^ adjuvanted intervention	NCT02334735
Myeloma	I	Completed	PD-L1	ISA-51 adjuvanted with montanide	NCT03042793
Myeloma	I	Recruiting	Multiple	Lenalidomide, durvalumab, and intervention with Hiltonol^®^ adjuvant	NCT02886065
NSCLC	I/II	Active	UCP2 and UCP4	ISA-51 adjuvanted with montanide	NCT02818426
Ovarian cancer	II	Completed	FOLR1	In addition to durvalumab	NCT02764333
Ovarian cancer	II	Terminated	FOLR1	Combined with GM-CSF	NCT02978222
Prostate cancer	I	Completed	BCL-X_L_	Combined with the drug montanide CAF09b	NCT03412786
Prostate cancer	I/II	Unknown	PSA	Intervention with GM-CSF or montanide ISA-51 adjuvant and hyperthermia, imiquimod, or RNA-based vaccine	NCT02452307
Prostate cancer	I/II	Completed	RHOC	Adjuvant: montanide ISA-51	NCT03199872
Prostate cancer	II	Completed	TERT	Montanide ISA-51 and imiquimod were used as adjuvants.	NCT02293707
Solid tumor	I	Completed	PPV	Combination of Hiltonol^®^ adjuvanted intervention and nivolumab	NCT02897765
Brain tumor	I	Recruiting	Multiple	GM-CSF and montanide ISA-51 adjuvanted intervention in combination with temozolomide	NCT03299309
Brain tumor	I	Withdrawn	PPV	Combined with Hiltonol^®^	NCT03068832
Gastroesophageal cancer	I/II	Active	HER2	When used in conjunction with cisplatin and 5-fluorouracil or capecitabine	NCT02795988
